# A sensitive and innovative detection method for rapid C-reactive proteins analysis based on a micro-fluxgate sensor system

**DOI:** 10.1371/journal.pone.0194631

**Published:** 2018-03-30

**Authors:** Lei Guo, Zhen Yang, Shaotao Zhi, Zhu Feng, Chong Lei, Yong Zhou

**Affiliations:** 1 Key Laboratory for Thin Film and Microfahrication of Ministry of Education, Research Institute of Micro/Nano Science and Technology, Shanghai JiaoTong University, Shanghai, China; 2 School of Physics and Electronic Engineering, Xinyang Normal University, Xinyang, China; Chung-Ang University College of Engineering, REPUBLIC OF KOREA

## Abstract

A sensitive and innovative assay system based on a micro-MEMS-fluxgate sensor and immunomagnetic beads-labels was developed for the rapid analysis of C-reactive proteins (CRP). The fluxgate sensor presented in this study was fabricated through standard micro-electro-mechanical system technology. A multi-loop magnetic core made of Fe-based amorphous ribbon was employed as the sensing element, and 3-D solenoid copper coils were used to control the sensing core. Antibody-conjugated immunomagnetic microbeads were strategically utilized as signal tags to label the CRP via the specific conjugation of CRP to polyclonal CRP antibodies. Separate Au film substrates were applied as immunoplatforms to immobilize CRP-beads labels through classical sandwich assays. Detection and quantification of the CRP at different concentrations were implemented by detecting the stray field of CRP labeled magnetic beads using the newly-developed micro-fluxgate sensor. The resulting system exhibited the required sensitivity, stability, reproducibility, and selectivity. A detection limit as low as 0.002 μg/mL CRP with a linearity range from 0.002 μg/mL to 10 μg/mL was achieved, and this suggested that the proposed biosystem possesses high sensitivity. In addition to the extremely low detection limit, the proposed method can be easily manipulated and possesses a quick response time. The response time of our sensor was less than 5 s, and the entire detection period for CRP analysis can be completed in less than 30 min using the current method. Given the detection performance and other advantages such as miniaturization, excellent stability and specificity, the proposed biosensor can be considered as a potential candidate for the rapid analysis of CRP, especially for point-of-care platforms.

## Introduction

Modern biomedical research requires accurate, sensitive, and rapid protein measurements to facilitate biomarker discovery [1], detection [2], monitoring of diseases [3], personalized medicine [4], and new drug development [5]. An important application involves measuring the levels of proteins in blood serum, which are biomarkers for disease diagnosis. To date, known protein markers exist for every common form of cancer as well as for a range of cardiac diseases [6]. An ability to assay the aforementioned biomarker-proteins is of profound value in early diagnosis, as well as in the monitoring of responses for therapeutic intervention [7]. Specifically, C-reactive proteins (CRP) are extremely important acute-phase proteins and are of particular interest because the presence of abnormal concentrations of CRP in blood is directly linked to various diseases [8, 9]. Recent studies indicated that CRP is directly involved in cardiovascular diseases [10, 11], and is the most reliable predictor of cerebral congestion [10] and myocardial infarction [11]. In fact, elevated CRP levels are a factor in the diagnosis of several fatal diseases, i.e., in the field of clinical application, CRP levels of 0.003–0.11 μg/mL can be used for the monitoring of coronary heart disease after surgery therapy and a CRP range from 0.005–3 μg/mL can be utilized for the diagnosis of potential risks of acute myocardial infarction. Moreover, Yanbaeva et al [12] indicated that the CRP-levels of 0.2–11.09 μg/mL are useful for the prediction of chronic obstructive pulmonary disease (COPD) risk. Furthermore, Reitz et al [13] found that a threshold of about 6 μg/mL can be served to assist in the diagnosis of cerebral small-vessel disease. Thus, high-sensitivity and wide linearity range of CRP measurement has the potential to provide valuable information for both medical diagnosis and treatment opportunities. Currently, immunoassays and related techniques are considered as major analytical methods with respect to the detection of CRP. The techniques typically include radioimmunoassay [14], enzyme immunoassay [15], chemiluminescence immunoassay [16], and fluorescence immunoassay [17]. Unfortunately, these methods tend to be either time-consuming or entirely lab-based, and therefore non-transferrable with respect to point-of-care platforms. This hinders the ability to perform large-scale disease screening. Consequently, alternative approaches with high sensitivity, rapid response, and easy manipulation are extremely necessary with respect to CRP analysis.

Magnetic biosensors based on detecting stray magnetic fields induced by immunomagnetic paricles have generated considerable research interest due to the potential for the development of high-performance bioanalyte assay systems. In addition to a low detection limit, a sensing system based on magnetosensors with magnetic bead-labels and sandwich immunoassays is easily manipulated, with a fast response time, and are miniaturized [18–21] when compared to other immunosensing techniques. This makes the technique a viable potential candidate for the rapid analysis of biomolecules and especially for point-of-care platforms. Several studies focused on developing a new generation of compact bioanalytical systems by incorporating magnetosensors. A number of magnetic biosensors including magneto-resistive sensors [18], spin-valves [19], hall sensors [20], giant magneto-impedance sensors [21], and superconducting quantum interference devices (SQUIDS) [22] have been proposed as biosensors for biomagnetic measurements.

Among magnetic detection methods, the application of the fluxgate principle constitutes one of the most important and well-developed sensing techniques. The fluxgate sensor exhibits several advantages when compared with other convention solid-state devices, such as a wide measuring range, high reliability, quick response time, cheap price, and high sensitivity that is comparable to the ultrasensitivity of a very expensive quantum-effect SQUIDS [23]. However, conventional fluxgate sensors are fabricated by mechanically winding coils on magnetic cores, and this results in numerous disadvantages, including large size, high weight, and high power consumption. This limits its application in bio-magnetic detection, in which compact size and portability are constantly required. Recently, significant progress has characterized micro-electro-mechanical system (MEMS)-based sensor manufacturing techniques with the rapid development of microelectronics technology [23, 24]. Micro-fluxgate sensors fabricated by using MEMS technology overcome the original shortcomings and also possess improved long-term stability and higher integration. Furthermore, the compatibility of the MEMS technique with the lab-on-chip technology can easily miniaturize the entire fluxgate sensing system into an integrated electronic chip for real-time magnetic monitoring, and this makes the MEMS-fluxgate sensor an ideal medium for the development of a point-of-care platform for on-site biomagnetic measurement.

In previous studies, Sun et al [25] and Yang et al [26] have introduced fluxgate sensor biosystems into the detection of Escherichia coli O157H:H7 and Alpha Fetoprotein (AFP), respectively. In these reported works, electroplated permalloy thin films were employed as the sensitive elements of the fluxgate sensor for the biomarker-labeled immunomagnetic beads analysis. However, due to the poor saturation induction density and low permeability of permalloy, it not only led to a low sensitivity of these systems, but also resulted in a poor linearity property for the biomolecules measurements. Moreover, the previous studies are based on the streptavidin-biotin immunoassays, where long-time incubation steps and multi-step magnetic separation procedures are always in demand. Therefore, these methods are generally time consuming and are inconvenient to operate. Furthermore, Song et al [27] and Bjerner et al [28] indicated that the streptavidin-biotin interactions suffer from the unavoidable interference of residual heterophilic antibodies, receptor antibodies, and biotin that are presented in the test samples, potentially leading to test errors and poor specificity property in practical applications. To address the limitations of recently reported works, an effective detection strategy based on fluxgate sensors and immunomagnetic beads-labels for the rapid sensitive analysis of CRP was explored in an effort to establish a bioanalyte monitoring system associated with the advantages of the MEMS-fluxgate sensor. In the current study, we described an ultra-sensitive detection method for rapid CRP analysis on a newly-developed amorphous ribbon-based fluxgate sensor by using antibody-conjugated immunomagnetic microbeads-labels and double polyclonal antibody pair immunoassays. As a departure from previous studies on this subject, we established a micro-fluxgate sensor based on Fe-based amorphous ribbon as the sensing elements with optimized structural parameters [23, 24]; this was done in order to achieve better sensitivity and wider linearity range properties in the CRP detection performance benefiting from the excellent ferromagnetic properties of Fe-based amorphous alloy [29]. Antibody-conjugated immunomagnetic microbeads were strategically utilized as signal tags to label the CRP through the specific conjugation of CRP to CRP antibodies. Separate Au film substrates were applied as immunoplatforms to capture and immobilize the CRP coupling with magnetic beads through double polyclonal antibody pairs. The detection and quantification of the immobilized CRP-bead complexes was implemented by detecting the stray field of CRP-labeled magnetic beads under an applied external magnetic field using the newly-developed micro-fluxgate sensor. The resulting system successfully achieved desirable sensitivity, stability, reproducibility, and selectivity. The minimum detection limit was as low as 0.002 μg/mL and was achieved with a linearity range of 0.002–10 μg/mL within the system, suggesting that the proposed biosystem is extremely sensitive. In addition to the extremely low detection limit, the sensing technique can be easily manipulated and responds quickly. The response time of the fluxgate sensor was less than 5 s, and the entire detection period for the CRP analysis, including the CRP samples immunoassay procedures and detection steps, can be performed in less than 30 min by using the system. On account of the detection performance and other advantages such as miniaturization, excellent stability and specificity, this fluxgate immunosensor can be considered as a potential candidate for on-site rapid analysis of CRP, particularly in point-of-care platforms.

## Experimental section

### The sensor fabrication process

**Fig 1:**
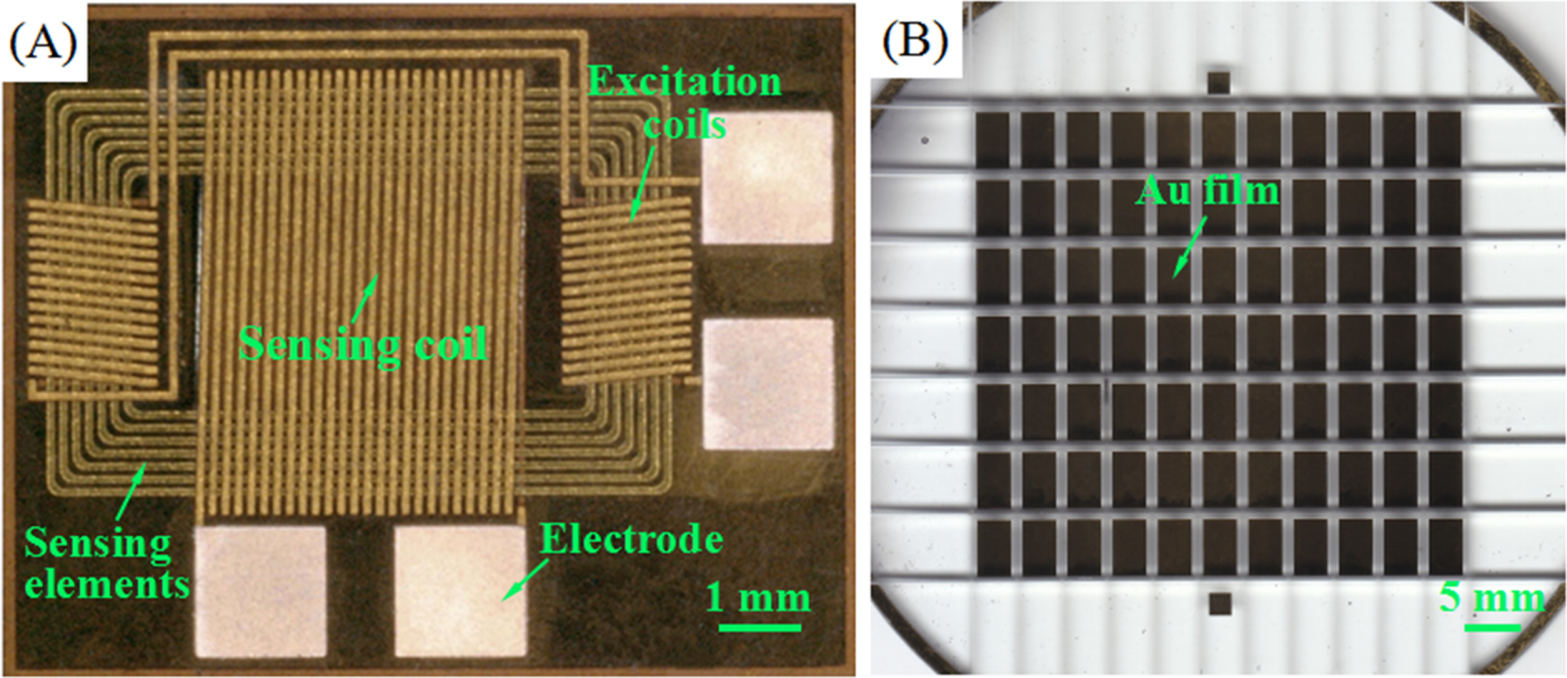
Photographs of the fabricated micro devices. (A) The MEMS-fluxgate sensor. (B) The Au film substrates (5×3 mm^2^).

The standard MEMS fabrication processes of thick photoresist-based UV lithography, electroplating, and micro assembly were used to fabricate the micro-fluxgate sensor. Details of the fabrication process are summarized in the Supporting Information section (S1 File). It is worth mentioning that the micro-sensors can be readily produced using MEMS technology in less than 15 h, and more than 60 sensors with different structural parameters can be obtained on one glass substrate during a single production period. In this study, the proposed fluxgate sensor contained a multi-loop magnetic core made of Fe-based amorphous ribbon (Fe_73.5_Cu_1_Nb_3_Si_13.5_B_9_) that acted as the sensing element, as shown in Fig 1A. Additionally, 3D solenoid copper coils (one sensing coil and two excitation coils) were used to control the magnetic sensitive elements. The sensing coils were placed vertical to the two excitation coils and in between them. Previous studies have shown the influence of structural parameters on sensor performance [23, 24]. Based on this, we designed the proposed sensor structure with the multi-loop sensing element, to obtain an optimal sensor sensitivity and better CRP detection performance. The sensitivity of the fabricated fluxgate sensor was also studied, as shown in the Supporting Information section (S2 File). The maximum sensitivity was 1.887 mV/μT at an excitation root mean square (RMS) current of 110 mA. Therefore, the same excitation RMS current condition was used in the experiments in this study, as discussed in the following sections.

The Au film substrates that were applied as the immunoplatforms for immobilization of the CRP coupled magnetic beads were fabricated by sputtering the Au-film on a separate glass wafer from the sensor substrate (S3 File). Subsequently, the wafer was sliced into several microchips in which each microchip possessed a rectangle-shaped Au film (5×3 mm^2^) on it, as shown in Fig 1B.

### Reagents

Bovine serum was purchased from Yuanmu Co. Ltd (ShangHai, China). Phosphate buffer tablets (pH 7.4) were purchased from Medicago AB (Uppsala, Sweden). Dynabeads®primary polyclonal CRP antibody coupling kits were purchased from Guidechem Co. Ltd (Hangzhou, China) and included uniform superparamagnetic beads with a diameter of 1.0 μm with polyclonal CRP antibodies coupled to the bead surface through epoxide-group binding. Human CRP and secondary mouse anti-human CRP polyclonal antibodies were purchased from Guidechem Co. Ltd (Hangzhou, China) as well. A phosphate-buffered solution (PBS, pH 7.4) was prepared with phosphate buffer tablets and deionized water. Furthermore, 11-mercaptoundecanoic acid was purchased from Aladdin Chemistry Co. Ltd. (Beijing, China), and 1-Ethyl-3-[3-dimet-hylaminoprop-yl] carbodiimide (EDC) hydrochloride was purchased from Aladdin Chemistry Co. Ltd (Beijing, China). Additionally, N-Hydroxysulfosuccinimide sodium salt (NHS) was purchased from Medpep Co. Ltd. (Shanghai, China). Deionized water was used in all the experiments.

### Antibody-modification on the Au film substrate

**Fig 2:**
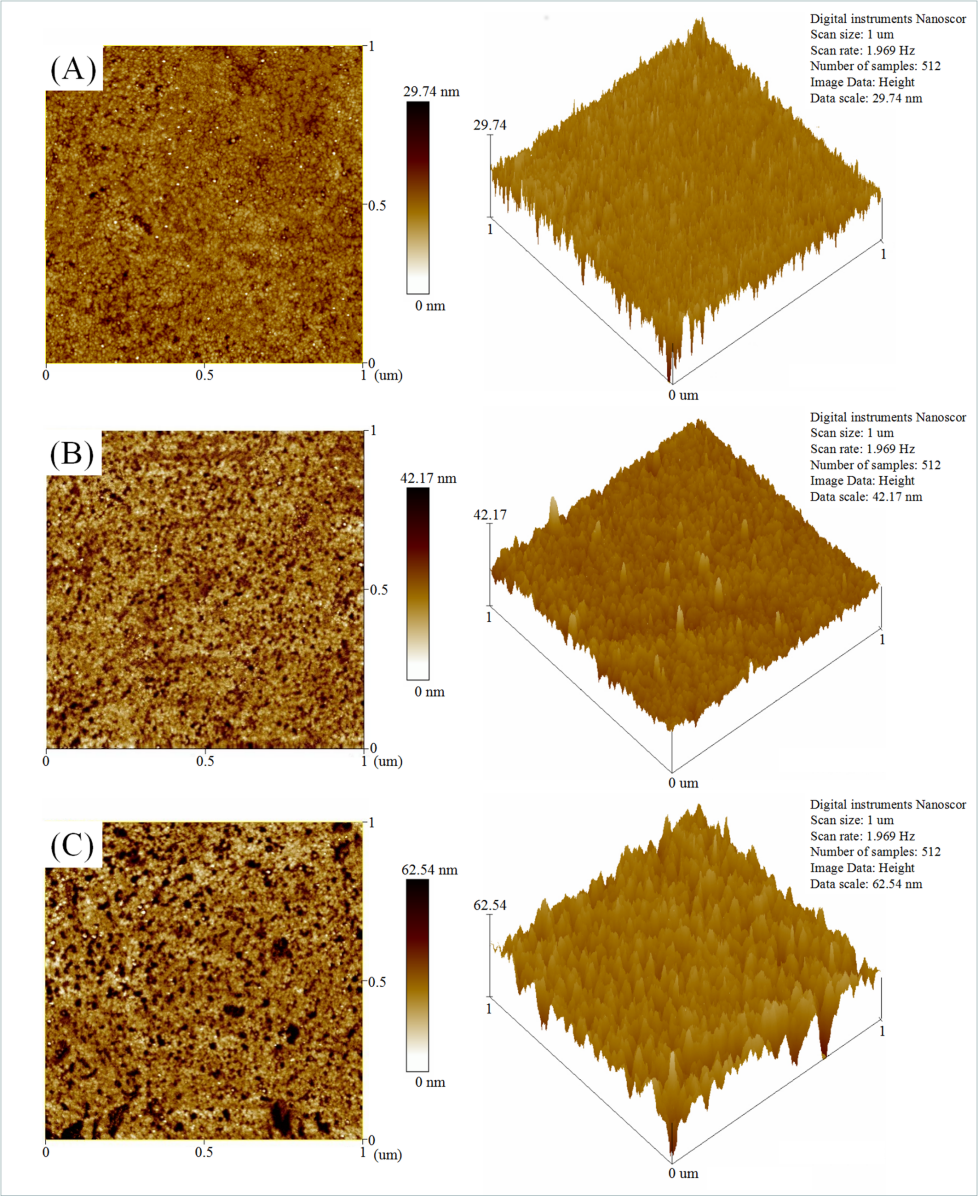
AFM images and roughness analysis of the modified Au film. (A) The blank Au film. (B) The Au film after 11-MUA modification. (C) The Au film after antibody modification.

After the Au film substrate was completely washed, 11-MUA that was used as a self-assembled layer was dropped on the Au film of the substrate to form 11-MUA molecular membranes through the Au-S covalent bond. Subsequently, the substrate was incubated in a mixture solution of 0.2 mol/L EDC and 0.05 mol/L NHS to activate the COOH group in 11-MUA at 4°C for 30 min, washed with PBS, and air dried. Next, the CRP polyclonal antibody was modified on the Au film of the substrate by incubating the wafer in 2 mg/mL antibody solutions for 30 min at 4°C. Atomic force microscope (AFM) was employed to characterize the Au film before and after modification as shown in Fig 2A–2C. The surface topography of the Au film changed clearly after it was modified with CRP antibodies. As shown by the roughness analysis, the surface roughness of the Au film increased significantly following the CRP antibodies modification. This indicated the successful modification of the CRP antibodies on the Au film. After the modification, the modified substrate was stored in a refrigerator at 4°C for further use.

### CRP samples preparation

The present limitation of our experimental conditions is that we do not have a qualified institution or hospital as a cooperator to supply us with clinical samples for human serum or plasma testing. Thus, to mimic real sample detection in complex media for evaluation of our system, the more commercially available and native CRP-free bovine serum was used to dilute the human CRP to different concentrations ranging from 0.002 to 10 μg/mL in this study.

### Immunoassay and detection methods of CRP coupled magnetic beads based on the system

**Fig 3:**
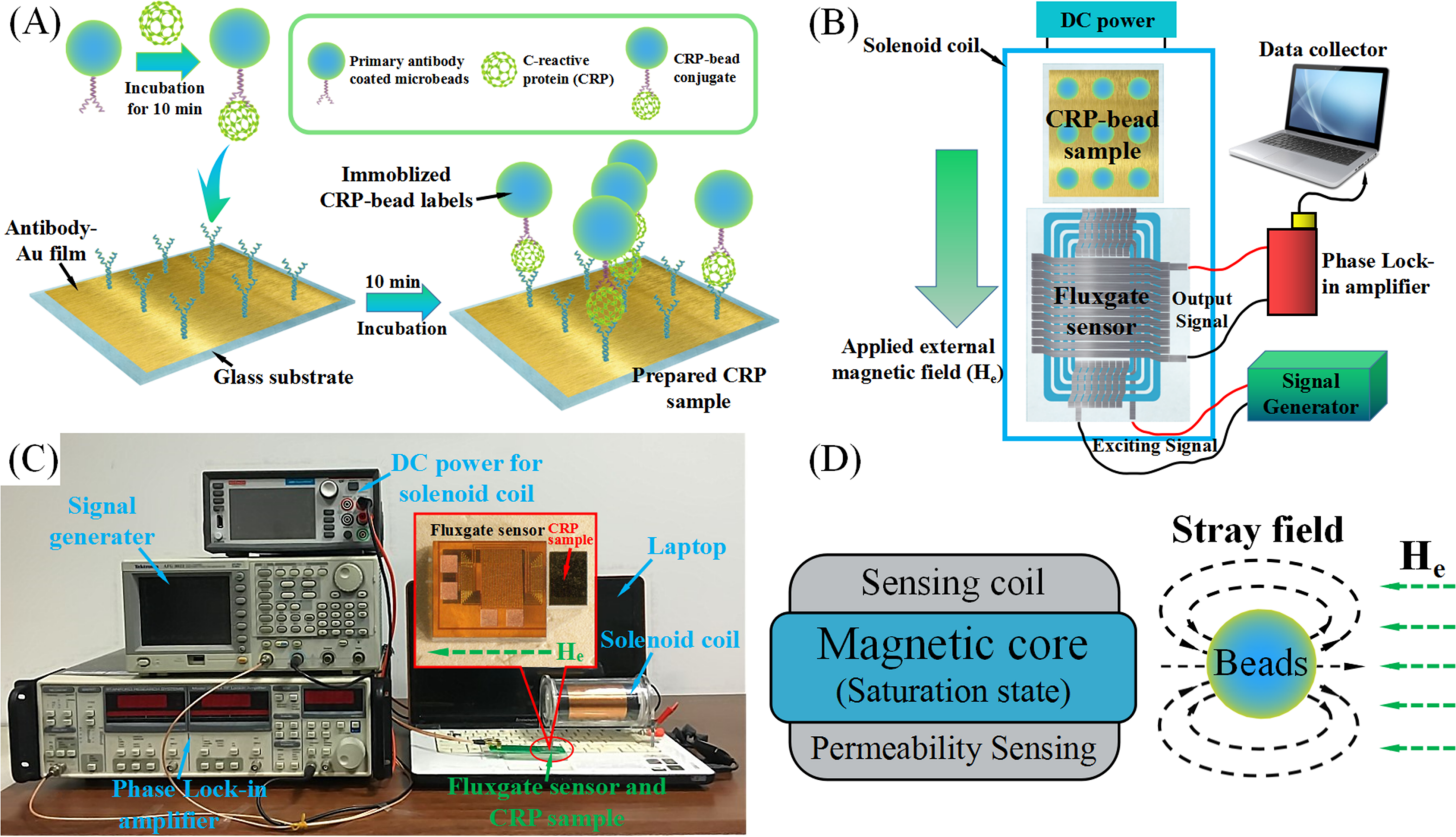
Immunoassay and detection method for CRP based on our system. (A) The immunoassay process of CRP. (B) Illustration of the detection method based on fluxgate sensor (C) The photograph of the fluxgate sensing circuit-system, the inlet shows the illustration of the relative position of the CRP sample in relation to the sensor. (D) CRP-labeled Dynabeads detection mechanism of the proposed system.

First, 5 μL of 300 μg/mL primary anti-CRP antibodies coated Dynabeads were added into 5 μL of the CRP samples and incubated at 37°C for 10 min for labeling the CRP with magnetic beads through the conjugation of CRP to primary polyclonal anti-CRP antibodies coated on the bead surface. A large amount of Dynabeads were used during the labeling process to ensure that most of the CRP responded well to the antibodies at the surface of the beads. After the labeling, the conjugated solutions were dropped on the modified-Au film substrate and incubated for 10 min at 37°C for specific containment of CRP-bead labels through the modified secondary antibodies on the Au film of the substrate. Finally, the substrate was washed with PBS solution to clean the non-target component and excess unlabeled magnetic particles to ensure the sensitivity of the system without background interference. Fig 3A shows a schematic illustration of the immunoassay process for CRP couplied with magnetic beads.

A second-harmonic-principle-based fluxgate sensing circuit-system consisting of an excitation and sensing loop was established for evaluating the CRP detection performance of our sensor, Fig 3B and 3C shows the illustration and photograph of the entire test system. The excitation circuits must ensure the magnetic sensitive element of sensor is working in a deep saturation state, resulting in a noticeable variation of magnetic induction of the sensing core attributable to the ferromagnetic properties of the soft magnetic core materials when an external magnetic field is tested [30]. The sensing circuits should be able to effectively pick up the variable magnetic induction from the sensing element through the sensing coil of sensor based on the second-harmonic-principle [31]. In this study, a signal generator (Tektronix AFG 3022, Tektronix Technology Co., Ltd.) was set to provide an alternating current (AC) signal to excite the sensing core into a saturation state through the excitation coil of our sensor. A phase lock-in amplifier (SR830-DSP, Stanford Research Systems Co., Ltd.) was utilized to pick up the second harmonic signal from the output of the sensing coil. The measuring results were collected with a laptop (Fig 3C). Within our bio-experiments, the Au film substrate with the captured CRP-bead labels was placed in front of the fluxgate sensor, along the longitudinal direction and against the magnetic sensitive core of our sensor for analysis (Fig 3B and the inlet of Fig 3C). Further, an external static magnetic field parallel to the longitudinal axis of the micro-sensor was provided by a solenoid coil to obtain better interval sensitivity of the sensor and the labeled Dynabeads samples tested were magnetized by the applied magnetic field, causing the beads to emit a stray field in which they were detectable. In the CRP detection phase, when the CRP-labeled Dynabeads sample was placed in front of the sensor and exposed to the applied external field (H_e_), a stray magnetic field was induced in the beads aligned to the H_e_ ascribed to the superparamagnetic property of the Dynabeads. This induced field was mainly unidirectional and opposite to the applied external field (Fig 3D). Consequently, the presence of the CRP-labeled Dynabeads could decrease the effective field experienced by the fluxgate sensor and potentially alter the effective magnetic induction of the Fe-based amorphous ribbon based sensing element. Thus changing the output response of our sensor. Through the difference in output response, we determined the level of the magnetic particles labeled CRP in the sample.

### Statistical analysis

Statistical analysis was performed using Graphpad Prism software to evaluate the significance among measurements. One-way ANOVA was used to determine significance between multiple groups. P-values <0.05 were considered as statistically significant.

## Results and discussion

### Characterization of the magnetic bead labeled CRP

**Fig 4:**
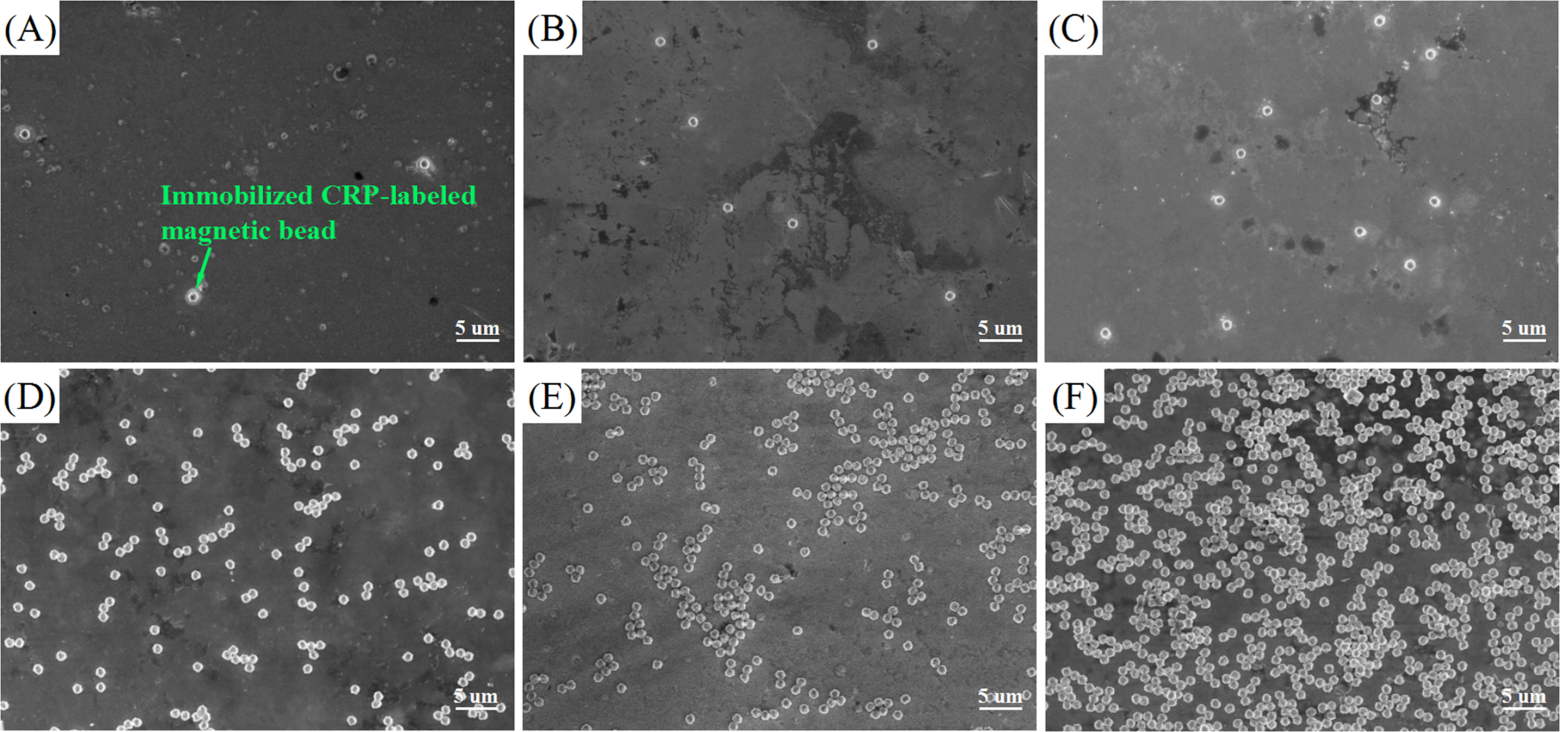
SEM images of immobilized CRP-bead labels of different CRP concentration samples. (A) 0.002 μg/mL. (B) 0.005 μg/mL. (C) 0.01 μg/mL. (D) 0.1 μg/mL. (E) 1 μg/mL. (F) 10 μg/mL.

Scanning electron microscopy (SEM) was utilized to characterize the immobilized CRP-bead labels on the modified-Au film substrate, as shown in Fig 4A–4F in which the quantities of Dynabeads increased clearly with increases in the CRP concentration. Thus demonstrating the Dynbeads-labeled CRP has been successfully immobilized onto surface of the Au film substrate.

### Detection sensitivity for CRP analysis

**Fig 5:**
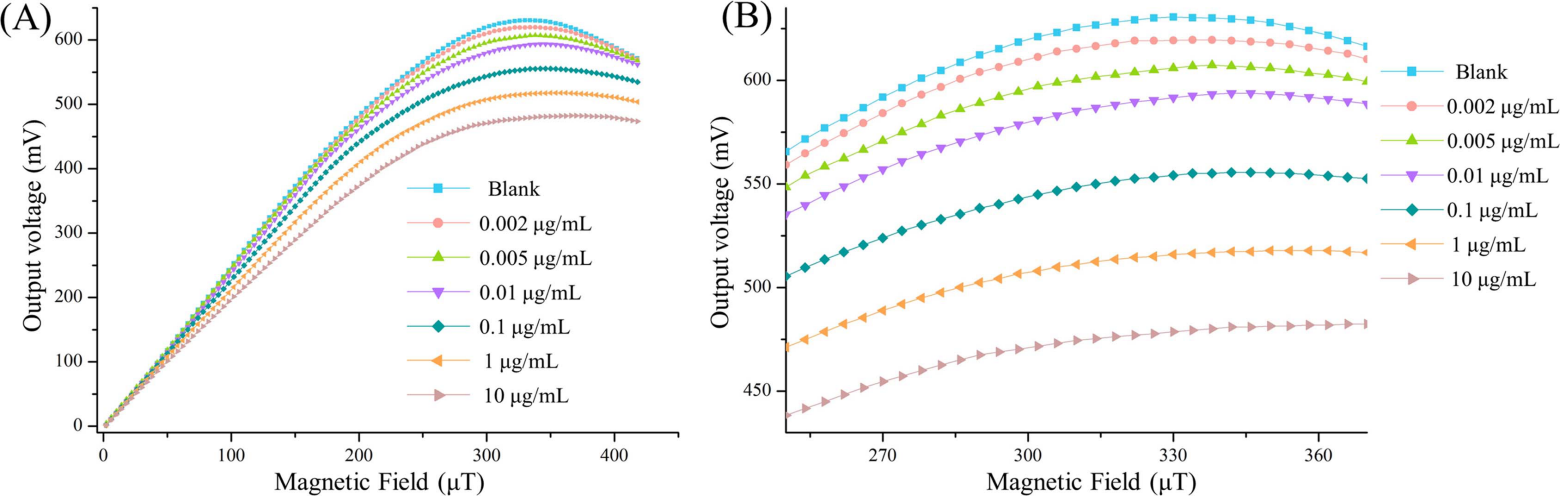
Detection sensitivity for CRP. (A) Full view of the output voltage towards different CRP concentration. (B) The partial enlargement of the field range corresponding to 250–370 μT.

Six concentration CRP samples (0.002 μg/mL, 0.005 μg/mL, 0.01 μg/mL, 0.1 μg/mL, 1 μg/mL, and 1 μg/mL) were used for assessing the detection sensitivity of our fluxgate sensor. For each concentration, five samples were prepared on five Au film microchips to evaluate the reproducibility of our method. In the present study, the CRP immunoassays were carried out on separate glass substrates from the sensor substrate, preventing chemical contamination or damage to our sensor during the assay procedure. Therefore, our sensor can be wash-free and conveniently reused. Consequently, the experimental data presented here were all collected from one-single fluxgate sensor. Fig 5 depicts the output voltage of various CRP samples subjected to the fluxgate sensing tests. There was an overall decrease in the output signal of the system attributable to the presence of CRP samples. The response time of the biosystem was less than 5 s. There were clear differences in the output signal at different CRP concentrations; however, it was also found that at a low external magnetic field range (less 200 μT), only minor variations were found in the output signal of the fluxgate sensor. This may be predominately caused by the low magnetic susceptibility of the magnetic beads under low external fields, thus resulting in a low-level stray field of the beads that is difficult to detect. Moreover, in the external magnetic fields >350 μT, the difference between the output voltage at different sample concentrations also decreased. This is mainly due to the magnetic flux density of our sensor becoming saturated under large enternal fields, making our sensor to become less sensitive to the tested stray field. Specifically, the maximum difference in the output signal of the fluxgate sensor can be found around the applied field of 330 μT and no interactions between any two curves were observed in the field range of 250–370 μT, indicating that each concentration of sample can be clearly distinguished by our method. A concentration of 0.002 μg/mL CRP was successfully detected by the system, thereby suggesting that the proposed biosystem is highly sensitive.

### Linearity for CRP analysis

**Fig 6:**
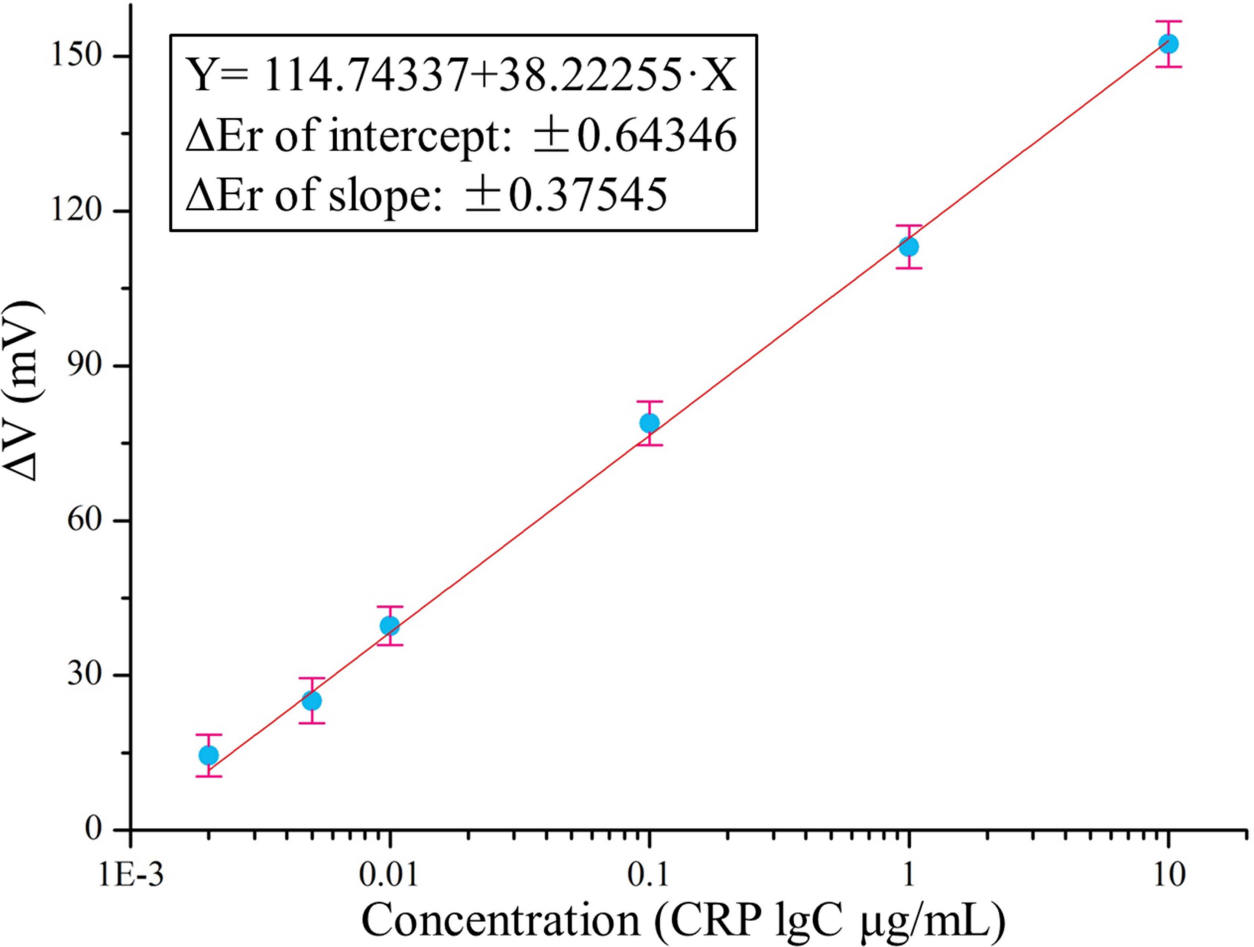
Linearity analysis. Linear range for the CRP detection with applied H_e_ = 330 μT.

Fig 6 shows the relationship between the CRP concentrations (lgC) and the corresponding output signal of the fluxgate sensing system. A straightforward linear relationship was observed between the CRP concentration (lgC) and ΔV of the biosystem at concentrations from 0.002 μg/mL to 10 μg/mL, and this can be used for further quantitative analysis. The linear regression equation is expressed as △V = 114.74337+38.22255·X (X = lgC, lg means log10, C represents the CRP concentration, μg/mL), with a correlation coefficient of 0.9913. Hence, △V represents the degree of output change, △V = (V_no_-V_sample_), where V_no_ and V_sample_ denote the output signals in the absence and presence of CRP, respectively. The results suggest that the developed sensing system is viable for the quantitative analysis of CRP samples.

### Reproducibility and stability

**Fig 7:**
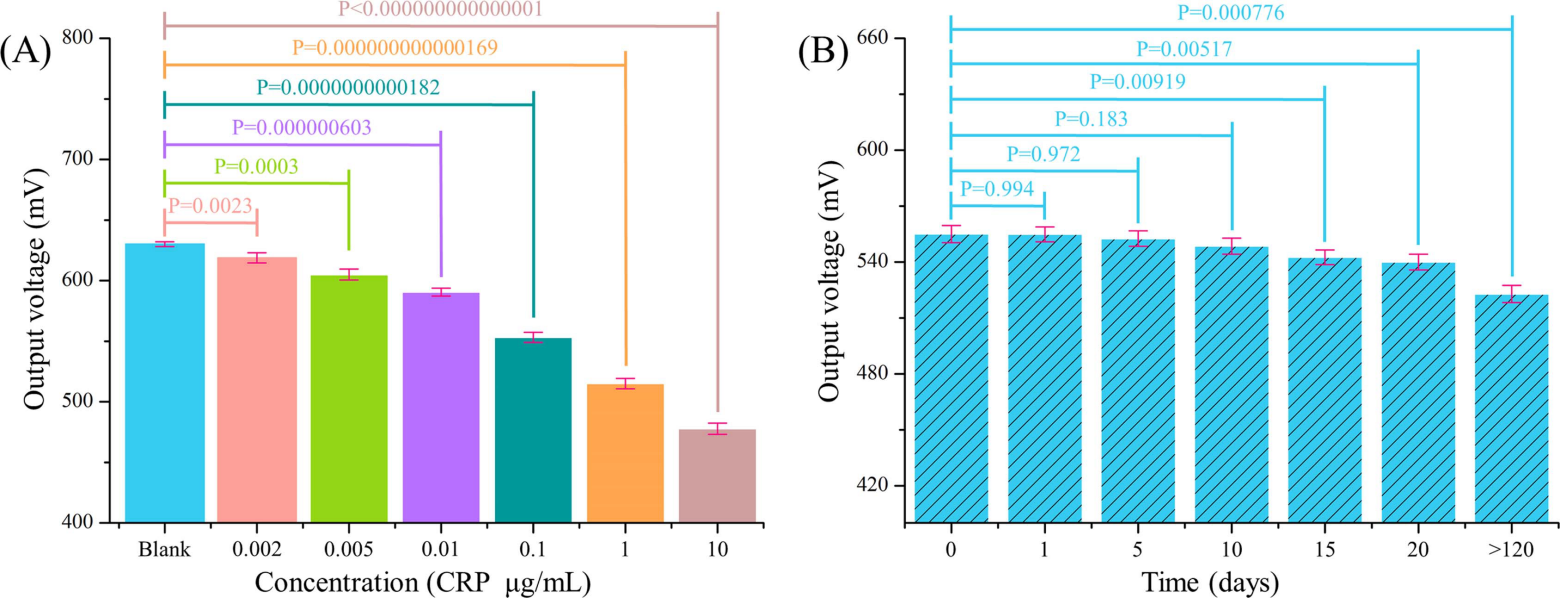
Reproducibility and stability test. (A) Reproducibility of the proposed biosystem to detect various CRP concentrations. (B) Stability test of the system with respect to 1 μg/mL CRP.

Five repetitive measurements for each concentration sample based on the system were performed under an external magnetic field of 330 μT to monitor the reproducibility of the system. Statistical analysis (One-way ANOVA) was performed using Graphpad Prism software to evaluate the statistically significant measurements, and P-values <0.05 were considered as statistically significant. As shown in Fig 7A, for different CRP concentration samples, the statistical analysis shows a series of P-values ranging from less than 10^−15^ to 0.0023, and the maximum P = 0.0023 was obtained at the CRP concentration of 0.002 μg/mL, demonstrating the statistical significance of the measuring results. The relative standard deviation (RSD) of the five independent assay results corresponded approximately to 0.958% and indicated the satisfactory reproducibility of the presented method. The capability test was repeated (5 times) after a gap, as shown in Fig 7B, to confirm the stability of our sensor. The results indicate that our sensor performance reveals a decreasing trend based on statistical significance (P = 0.00919) after 15 days. Since the current fluxgate sensor has not been fully encapsulated via an electronic packaging technology–potentially leading to oxidation of the sensor’s surface electrode and top coils when exposed to an air atmosphere for a long-time–this may be the main reason for the decrease in sensor performance after storage. However, our biosensor still retained approximately 94.21% of its initial response after being stored for more than 120 d, indicating the excellent stability of our sensor.

### Specificity

**Fig 8:**
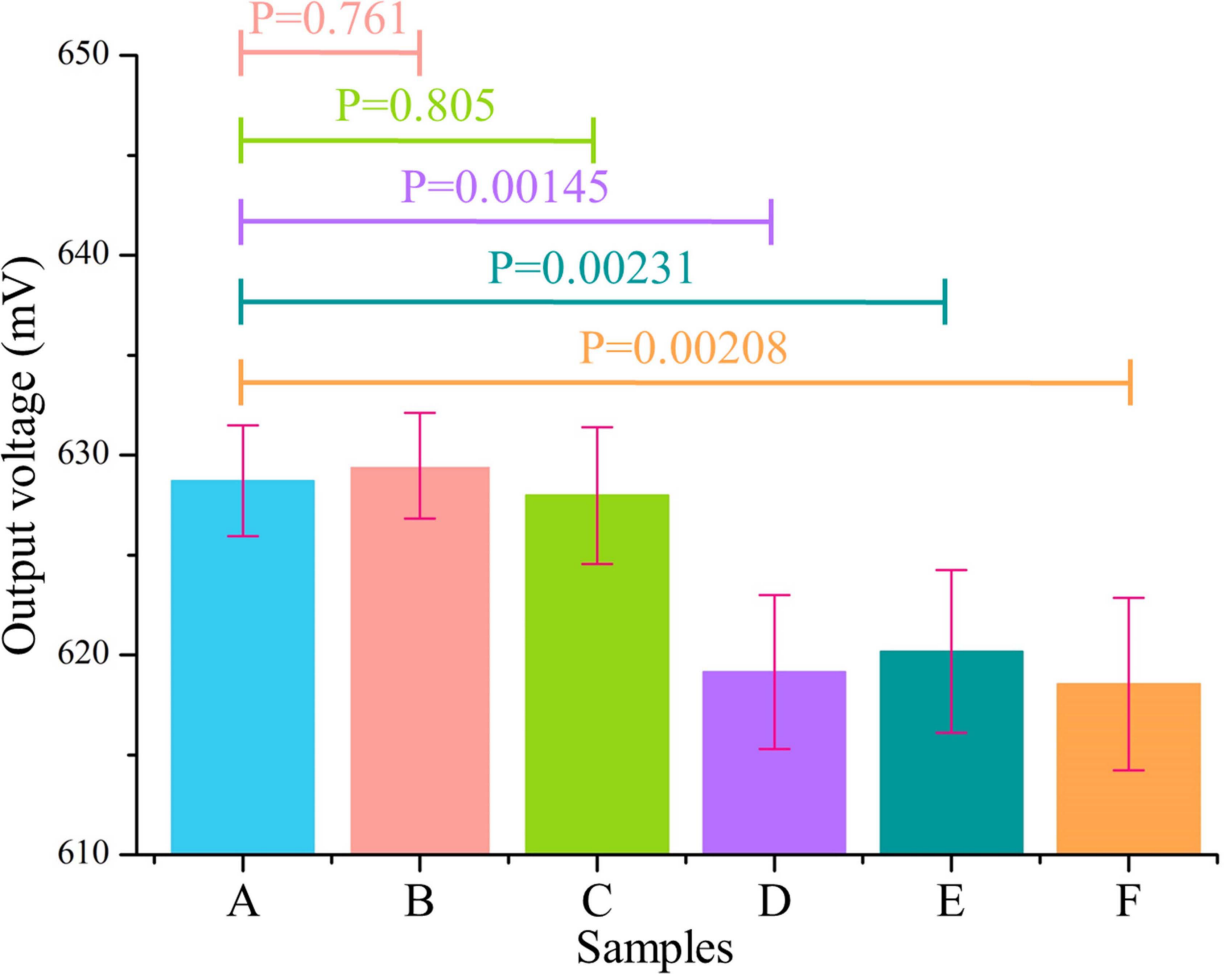
Specificity investigations of the biosystem. (A) Blank. (B) AFP (0.02 μg/mL). (C) CEA (0.02 μg/mL). (D) CRP (0.002 μg/mL). (E) CRP (0.002 μg/mL) +AFP (0.02 μg/mL). (F) CRP (0.002 μg/mL) + CEA (0.02 μg/mL).

In addition to the sensitivity, specificity is a key factor to assess the practicality of immunosensors. The specificity of the immunosensing system for CRP detection was tested via interfering proteins, such as alpha fetoprotein (AFP) and carcinoembryonic antigen (CEA), in addition to the CRP. The assay was performed using the same experimental procedures as described above, and the ratio between the target analyte and the interfering agent was 1:10. As shown in Fig 8, the output voltage was nearly the same as that of the blank sample when the system was only subjected to the interfering agent, and the statistical analysis shows P-values of 0.761 and 0.805 for the interfering proteins AFP and CEA, respectively, indicating the non-significance on statistical analysis for the interfering agent. There was a significant decrease in the output signal of the system when CRP was introduced with the aforementioned interfering agents (P = 0.00231 for CRP+AFP, P = 0.00208 for CRP+CEA), and no significant difference was found when compared with that of the CRP alone (P = 0.00145). The results indicate the selective binding of CRP to the CRP antibody. Thus, the system possesses a highly selective and specific detection capability with respect to CRP antigens.

Furthermore, a comparative study was performed with respect to the performance of the recently proposed immunosensor in CRP detection with other methods (as shown in Table 1). The results indicated that our fluxgate sensing system is highly sensitive and has a wider linear range when compared with those in recently reported studies. Conversely, the detection limits of a reported photothermal system (PTB) [32], and a metal-organic framework nanomaterials-based (MOF) electrochemical immunoassay (ECL) technique [33], or the Molybdenum-disulfide-polyaniline-gold nanoparticles (MoS_2_-PANI-GNPs)-based immunosensor [34], were significantly lower than that in the current study. However, the PTB sensing technique is based on the photothermal effect, and it therefore needs to be performed under strict temperature monitoring. Thus, it requires sophisticated instrumentation and is entirely lab-based. The MOF-ECL method or MoS_2_-PANI-GNPs-based immunosensor all require a series of very complicated biochemical reactions and immunization procedures to improve immunoassay efficiency for CRP coupling that demands the presence of professional and technical personnel for operation and is very time consuming. The proposed method is simple, easily manipulated, lab-free, and responds quickly. Specifically, the response time of the sensing system is less than 5 s, and the entire CRP detection period, including CRP samples immunoassay procedures and detection steps, can be accomplished in less than 30 min by using the described method. In addition to the detection performance of our system (i.e., sensitivity, linearity, and response time), the proposed fluxgate immunosensor also exhibited excellent stability and specificity. Moreover, since the CRP immunoassays were carried out on a separate glass substrate from the sensor substrate in the current study, this prevents chemical contamination or damage to our sensor during the assay procedure, signifying our fluxgate sensor is wash-free and conveniently reusable. However, despite the excellent overall performance of the proposed fluxgate sensor, this method also suffers from several problems: (i) Although the fluxgate sensor is miniaturized by the MEMS technology, the entire signal processing circuit-loops are still relatively complicated and are not integrated with the fluxgate sensor, thus limiting the portable property of the complete sensing system. In recent works, Baschirotto et al [35] and Qian et al [36] have already reported the CMOS-based readout-circuit chips for fluxgate sensors, which is fully integrated and highly portable (less than 4×2 cm^2^). However, due to current limitations in our experimental conditions, we temporarily lack the capability to establish such integrated signal processing circuit-chip for our sensors at this moment, this may be the main defect in our presented fluxgate sensor system. (ii) Another drawback potentially impacting our biosystem in practical applications is that, due to the magnetic induction saturation principle of fluxgate sensors, the presented sensors are not able to detect CRP samples with overhigh concentrations (dozens to hundreds, μg/ml). However, such a high CRP concentration assay is very useful for the determination of inflammation infection in clinical applications, possibly limiting some application field of our sensor for clinical diagnosis.

**Table 1:** Comparison of different immunosensors for CRP detection.

Materials, Methods	Linearity Ranges	Detection Limit	Detection Time	Ref.	Advantages	Disadvantages
Photothermal biosensor (PTB)	0.1–100 ng/mL	0.0001 μg/mL	More than 1 h	[32]	Most sensitive, miniaturization.	Requires sophisticated instrumentation, entirely lab-based, poor linearity property.
Metal-Organic-Framework-Nanomaterial-based immunosensor, ECL	1–400 ng/mL	0.0002 μg/mL	More than 24 h	[33]	Ultra-sensitive, excellent stability and specificity.	Very complicated and require technical professionals to operate, long detection time.
Molybdenum-disulfide-polyaniline-gold-particles-based immunosensor	0.2–80 ng/mL	0.0002 μg/mL	More than 12 h	[34]	Ultra-sensitive, excellent specificity.	Complicated and require technical professionals to operate, long detection time.
Immunofluorescent Nanospheres, LFT	0.025–1.6 μg/mL	0.0039 μg/mL	20 min	[37]	Short detection time, simple, excellent stability and specificity.	Relatively poor linearity range.
Nanocrystalline diamond sensor	10–20 μg/mL	10 ug/mL	1.5 h	[38]	Simple, excellent stability and specificity.	Low detection limit and poor linearity range, expensive.
Vertical flow immunoassay (VFA) biosensor	0.01–1 μg/mL	0.01 μg/mL	2 min	[39]	Sensitive, most rapid analysis.	Poor linearity property and require technical professionals to operate, disposable.
3D paper, VFAs	0.005–5 μg/mL	0.005 μg/mL	15 min	[40]	Short detection time, highly sensitive, wide detection range, miniaturization.	
Impedimetric, diamond-based biosensor	1.1–110 μg/mL	1.1 μg/mL	More than 1 h	[41]	Excellent stability and specificity.	Relatively poor sensitivity and expensive.
Optimised-gold-electrodes-chemical impedimetric biosensor	0.055–5.5 μg/mL	0.021 μg/mL	30–60 min	[42]	Sensitive, cheap, reusable.	Poor linearity property and stability, relatively poor reproducibility.
Carbon nanofiber based biosensor	0.05–5 μg/mL	0.011 μg/mL	1 h	[43]	Sensitive and cheap.	Poor linearity property and specificity.
Current work	0.002–10 μg/mL	0.002 μg/mL	Less than 30 min	-	Highly sensitive and short detection time, good stability and specificity, wash-free and reusable.	Limited portability due to the relatively complicated signal processing circuit.

## Conclusion

In this study, a sensitive and rapid assay technique based on a micro-MEMS-fluxgate sensor was designed, fabricated, and tested for the quantitative analysis of CRP. Within the proposed biosystem, we established a micro-fluxgate sensor based on Fe-based amorphous ribbon as the sensing elements with optimized structural parameters, in order to achieve better sensitivity and wider linearity range properties in the CRP detection performance benefiting from the excellent ferromagnetic properties of Fe-based amorphous alloy. Antibody-conjugated immunomagnetic microbeads were strategically utilized as signal tags to label the CRP through the specific conjugation of CRP to CRP antibodies. Additionally, separate Au film substrates were applied as the immunoplatforms to specific capture the CRP-bead conjugates through the modified self-assembled polyclonal CRP antibody layers combined using classical sandwich immunoassays. Subsequently, the detection and quantification of captured CRP-bead labels was implemented by detecting the CRP labeled magnetic beads using the newly-developed micro-MEMS-fluxgate sensor. The resulting system was able to detect CRP to a sufficient degree of sensitivity, stability, reproducibility, and selectivity. A minimum detection limit as low as 0.002 μg/mL of CRP was achieved, with a linearity range of 0.002 μg/mL-10 μg/mL, thereby suggesting that the proposed biosystem exhibits high sensitivity. The operation of CRP detection methods reported by extant studies always requires sophisticated instrumentation, professional and technical personnel, and is time-consuming. In contrast, the proposed method is simple, readily manipulated, sensitive, and capable of responding quickly. The response time of the presented sensing system was less than 5 s, with respect to the entire CRP detection period, including CRP samples immunoassay procedures and detection steps were performed within 30 min. It is expected that the results of this study will significantly enhance the practical applications of fluxgate systems in bio-magnetic-field sensing.

## Supporting information

S1 File Fabrication process of the micro-fluxgate sensor. (DOC)

S2 File Sensitivity characterization of the fabricated fluxgate sensor. (DOC)

S3 File Fabrication process of the Au film substrate. (DOC)
