# Supplementary material for: A sensitive and innovative detection method for rapid C-reactive proteins analysis based on a micro-fluxgate sensor system
Source: PLoS One. 2018 Mar 30;13(3):e0194631. doi: 10.1371/journal.pone.0194631 (PMC5877836; doi:10.1371/journal.pone.0194631)
Supplement: S1 File — (DOC) [file pone.0194631.s001.doc]

The fabrication of the micro-fluxgate sensor was performed on a 5 inches circular glass wafer. Figure A1-A9 shows the MEMS-based fabrication process of the micro sensor. The [fabrication](../../../../C:/Documents%20and%20Settings/Administrator/Local%20Settings/Application%20Data/Yodao/DeskDict/frame/20150830222619/javascript:void(0)%3B) process is as follows: (1) Cr/Cu seed layer was sputtered on the glass substrate for electroplating, and positive photoresist was spun on the seed layer which was patterned by ultraviolet lithography with the templet of bottom coil. Then, 30 um Cu film was electroplated in the photoresist mold to act as the bottom coil. (2) After the bottom coil was completed, positive photoresist was spun and patterned with the templet of vias. Vertical Cu cylinder was electroplated in the photoresist mold to act as the Cu vias to connect the top coil. (3) Then the seed layer was removed by reactive ion etching after the photoresist was eliminated with acetone. (4) Polyimide was spun on the wafer and baked at 250 °C in vacuum for 2 h for solidification, here polyimide was used for electrical insulation in order to isolate the sensing elements from the bottom Cu coil. Then the polyimide was etched by reactive ion etching to expose the Cu vias. (5) Then, another Cr/Cu seed layer was deposited on the surface and Metglas@2605 Fe-based magnetic cores (Fe_73.5_Cu_1_Nb_3_Si_13.5_B_9_), with a thickness of 10 um, were stuck onto the wafer. (6) Positive photoresist was spun and patterned with the templet of vias. The Cu vias was then electroplated to reach a height over the magnetic core. Afterwards the Cr/Cu seed layer was removed by reactive ion etching after eliminating the photoresist. (7) Polyimide was spun on the wafer again, and baked at 250°C in vacuum for 2 h for the purpose of isolating the sensor core from the top coils. Then the polyimide was etched by reactive ion etching to expose the Cu vias. (8) After coating the Cr/Cu seed layer on the wafer again, the positive photoresist was spun on the seed layer and patterned with the templet of top Cu coil, afterwards top Cu film with thickness of 30 um was electroplated in the photoresist mold to form the top Cu coil (9) Finally, the whole sensor was obtained after removing the photoresist and seed layer. The entire fabrication process can be completed by using MEMS technology in less than 15 h, and more than 60 sensors with different structural parameters can be obtained on one glass substrate during a single production period (as shown in Figure A10).

*
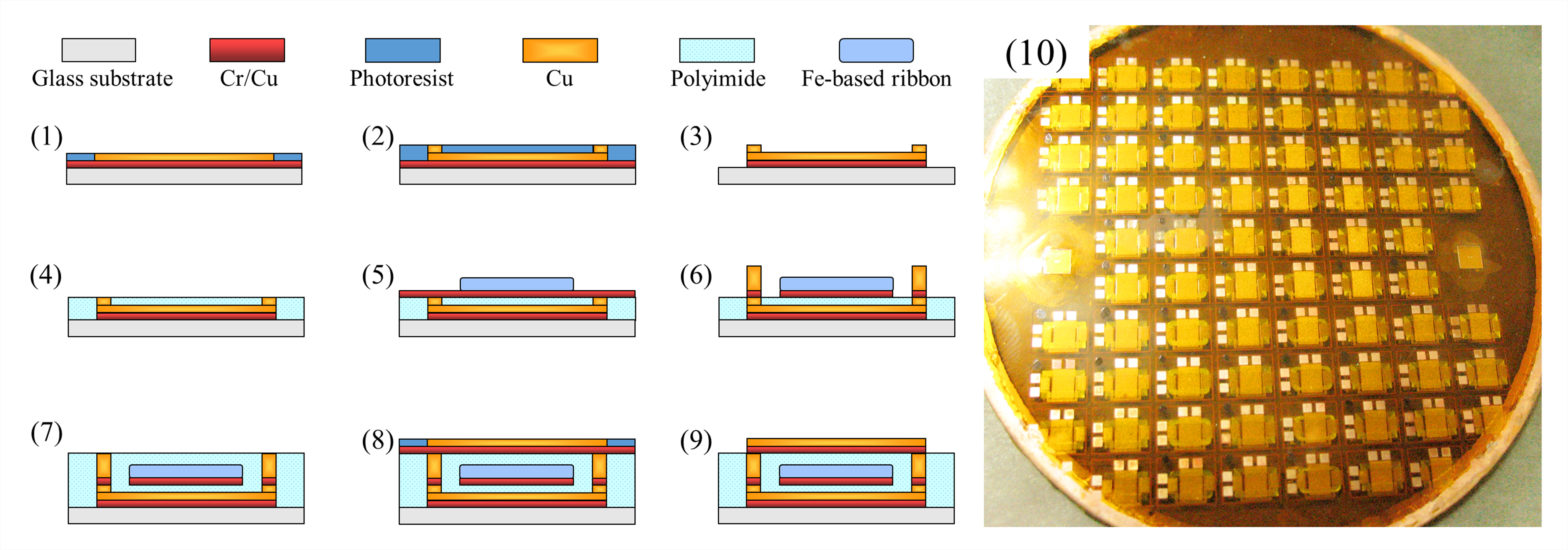
*

**Figure A. Fabrication process of the micro-fluxgate sensor.** (1-9) The detail fabrication procedure of the fluxgate sensor. (10) Photograph of the fabricated sensor substrate.
