# Supplementary material for: A sensitive and innovative detection method for rapid C-reactive proteins analysis based on a micro-fluxgate sensor system
Source: PLoS One. 2018 Mar 30;13(3):e0194631. doi: 10.1371/journal.pone.0194631 (PMC5877836; doi:10.1371/journal.pone.0194631)
Supplement: S2 File — (DOC) [file pone.0194631.s002.doc]

Sensitivity of the micro fluxgate sensor needs to be characterized as it determines the minimum detection limit of the system. The intensity measurement of external magnetic field (H_e_) was first performed to the fabricated micro fluxgate sensor without any samples. Figure B exhibits the output voltages of the detection system with the excitation coils being driven by AC currents with varying effective values.

According to Figure B, the following parameter was selected for the detection experiments: an excitation root mean square (RMS) current 110 mA, which was proven to be an optimal condition for the detection sensitivity of the fabricated sensor. Under this condition, a maximum sensitivity of 1.887 mV/uT was achieved. The same excitation conditions, i.e. excitation RMS current of 110 mA, were used in the following experiments in this paper.


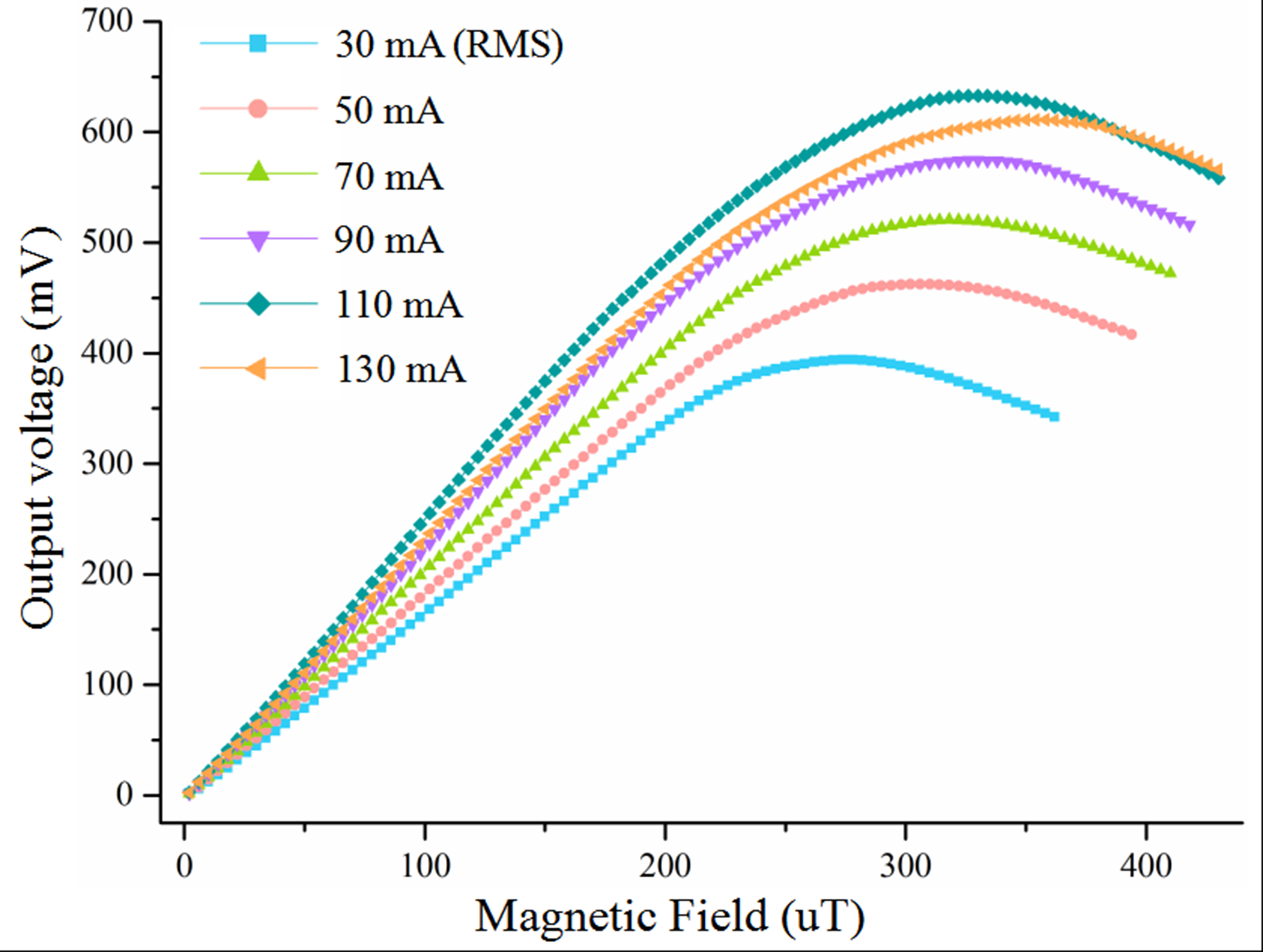


**Figure B. Sensitivity characterization of the fabricated fluxgate sensor.** Relationship between the sensor sensitivity and excitation current.
