# Supplementary material for: A sensitive and innovative detection method for rapid C-reactive proteins analysis based on a micro-fluxgate sensor system
Source: PLoS One. 2018 Mar 30;13(3):e0194631. doi: 10.1371/journal.pone.0194631 (PMC5877836; doi:10.1371/journal.pone.0194631)
Supplement: S3 File — (DOC) [file pone.0194631.s003.doc]

The entire fabrication process comprises the following steps (Figure C): (1) An Au layer of 300 nm was deposited on a 3 inches glass wafer. (2) A photoresist layer was spun on the Au layer and patterned to several rectangles with sizes of 5 mm x 3 mm by thick photoresist-based lithography. (3) The uncovered part of the Au layer was removed by chemical wet etching. (4) The whole sample was obtained after the photoresist was removed by acetone.


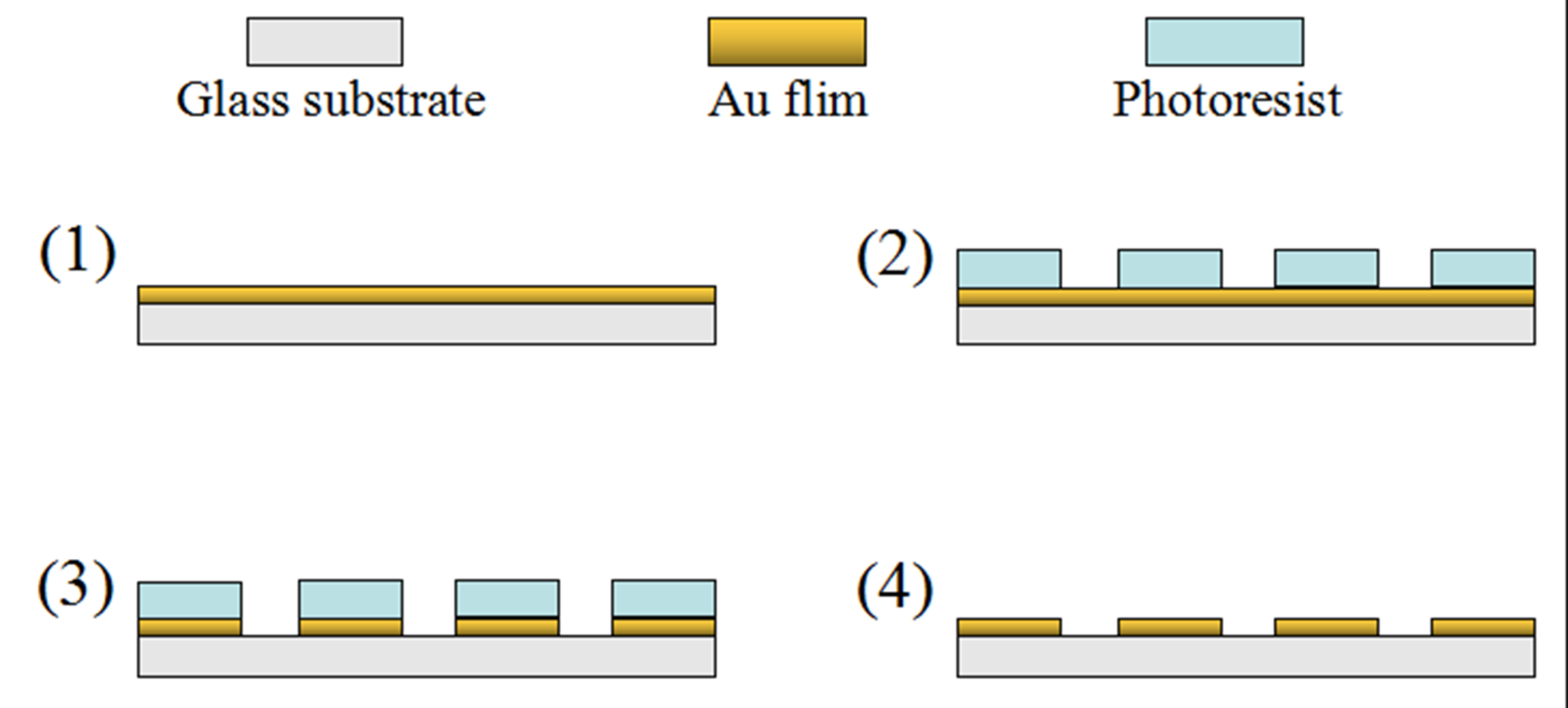


**Figure C.** **Fabrication process of the Au film substrate.** (1-4) The detail fabrication procedure of the Au film substrate.
